# Downregulation of Angiogenesis Factors, VEGF and PDGF, after Rapid IgE Desensitization and Oral Immunotherapy in Children with Food Allergy

**DOI:** 10.1155/2014/372567

**Published:** 2014-06-03

**Authors:** Paloma Poza-Guedes, Yvelise Barrios, Victoria Fuentes, Andres Franco, Inmaculada Sánchez-Machín, Elena Alonso, Ruperto González Pérez, Sonsoles Infante, Lydia Zapatero, Víctor Matheu

**Affiliations:** ^1^Consulta de Alergia Infantil, Unidad de Alergología-Norte, Hospital del Tórax/Ofra, CHUNSC, Ofra, Spain; ^2^Immunology, Hospital Universitario de Canarias, La Laguna, Spain; ^3^Consulta de Alergia Infantil, Hospital Infantil Gregorio Marañon, Madrid, Spain; ^4^Department of Clinical Sciences-Division IV, Lund University, Sweden

## Abstract

*Background*. Angiogenesis has a key role in several conditions and is regulated by several factors such as the platelet-derived growth factor (PDGF) or the vascular endothelial growth factor (VEGF). The goal of this study was to investigate the possible role of PDGF and VEGF in a group of patients with severe food allergy. *Methods*. We design a prospective longitudinal study (*n* = 30) with patients with persistent cow's milk proteins (CMP) allergy. After achieving a CMP rush desensitization protocol, a clinical followup including SPT and blood samples to determine sIgE, protein levels, PDGF, and VEGF-A and a panel of the most representative Th_1_, Th_2_, T_reg_, and Th_17_ cytokines were also monitored. *Results*. Baseline levels of PDGF and VEGF in the CMP allergic patients (1170 pg/mL and 253 pg/mL) were different compared to those nonallergic CMP control subjects (501 pg/mL and 108 pg/mL). Both PDGF and VEGF were significantly downregulated (*P* < 0.05) 6 months after completion of the CMP desensitization process and remained significantly decreased 12 months later. *Conclusion*. The present study shows a significant increase of PDGF and VEGF in anaphylaxis suffering children compared to a control group. Interestingly, both VEGF and PDGF were significantly downregulated after completing a full CMP rush IgE desensitization.

## 1. Introduction

Food allergy is a growing problem in developed countries and a recent meta-analysis in US patients found an increase of almost 20% in the current prevalence of food allergy over the past decade [1]. Although food allergy patients are generally expected to outgrow their condition, it may take longer than previously believed [2]. Regrettably, despite strict dietary avoidance, food allergy still continues to be the leading cause of anaphylaxis [3] among the pediatric patients with a profound impairment in their quality of life [4]. These are only some of the encouraging reasons that lead an increasing number of investigators to develop novel therapeutic approaches (i.e., food desensitization and/or food immunotherapy) to a better management of this health condition.

Angiogenesis, the blood vessel formation from already-existing blood vessel tissue, is regulated by several factors which have received an increasing interest because of their role in tumor growth and metastatic spread. One of the factors, the platelet-derived growth factor (PDGF), has been linked to several diseases such as malignant diseases and atherosclerosis or fibrosis [5]. PDGF is synthesized and released by platelets upon activation and smooth muscle or endothelial cells. PDGF can also be released by activated macrophages [6]. A different angiogenic factor, the vascular endothelial growth factor (VEGF), is described as the most potent proangiogenic factor regulating endothelial cells, increasing vascular permeability, promoting cell migration, and inhibiting apoptosis [7]. VEGF has been identified as an important target of cancer therapy [8]. The block of endothelial cell VEGF activity inhibits tumor angiogenesis, normalizes tumor vasculature, facilitates improved chemotherapy delivery, and prevents the recruitment of progenitor cells from the bone marrow [8].

However, vascular remodeling is associated with increased vascular permeability; it is also a key feature in the pathogenesis of many chronic inflammatory diseases [9] including lung disorders [10] such asthma [11, 12]. It has been also shown to have an essential role of nitric oxide in VEGF-induced, asthma-like angiogenic, physiologic responses in the lung and some features of phenotype as mucus [10], and it is even linked with atopic dermatitis [13]. In addition,* in vitro* studies have also proved that IgE could induce the VEGF production in mast cell lines [14].

The goal of this study was to investigate the role of a panel of the most representative cytokines of the Th_1_, Th_2_, T_reg_, and Th_17_ and the angiogenic factors PDGF and VEGF in a group of patients with severe allergy—that is, anaphylaxis—after the ingestion of cow's milk protein (CMP), the most prevalent food allergy in the early years of life. In addition, we have monitored the variation in these factors before and after the fulfillment of a standardized clinical intervention (i.e., CMP rush desensitization) with a marked clinical outcome in the course of the persistent disease.

## 2. Methods

### 2.1. Study Design and Population

We design a prospective longitudinal series study of 30 pediatric patients with a diagnosis of persistent cow's milk proteins (CMP) severe allergy. All children included in the active study group complained with anaphylaxis episodes [15] after the ingestion of CMP and frequent nonscheduled visits to the Emergency Room (ER), despite a correct CMP restrictive diet. These patients have positive skin prick tests and high levels of specific IgE (sIgE) antibodies to CMP, with a distinct immunological phenotype showing low levels of the granule neutral proteases such as monocyte chemotactic protein 1 (MCP-1) and macrophage inflammatory protein 1 *α* (MIP-1*α*), as previous described [15, 16]. A matched group of 30 pediatric patients with no food allergy and no dietary restrictions fulfilled the control group. The informed consent was obtained from parents or guardians and the CMP protocol has been previously approved by the Hospital Ethics Committee (PI CHUNSC: 35/11).

### 2.2. Clinical Intervention

Those patients with CMP anaphylaxis underwent a two-step standardized protocol starting with a rapid CMP desensitization at the Pediatric Critical Care Unit and followed by a subsequent oral CMP immunotherapy treatment (OIT) at the Allergy Office to achieve tolerance to the CMP contained in a glass of milk (250 mL) in 6 weeks as described elsewhere [15]. Allergic patients and their tutors were trained and fostered to recognize and treat the early symptoms of allergic reactions (oral antihistamines or corticosteroids), including anaphylaxis [17] with epinephrine autoinjector device, while kept in permanent telephone contact with the ER medical staff.

### 2.3. Serum Sample Collection and Measurement of Protein and Factors

A clinical followup including sIgE and blood samples for the measurement of protein levels was collected and monitored at different timelines (baseline and 6 and 12 months) for every patient. After venopuncture, serum was obtained after centrifugation and stored at −80C until the assay experiment was completed. All* in vitro* experiments were done at the same assay to avoid any* in-house* differences. Specific IgE antibodies against CMP, casein, *α*-lactalbumin, and *β*-lactoglobulin were determined by using enzimoimmunoassay (Phadia, Uppsala, Sweden). Along with PDGF and VEGF-A, a panel of the most representative cytokines of the Th_1_, Th_2_, T_reg_, and Th_17_ response was determined in plates by using a designed immunoassay analysis. Very briefly, the wells in the plate were soaked and a diluted capture bead solution was added. Then it was vortex and sonicated. Then, after washes, an incubation buffer was added to every well and standards were added into designated wells. Samples were added with assay diluents. The plate was then covered and incubated for 2 hours at room temperature on a shaker. Then, after washes, the prepared biotinylated detector antibody diluting in biotin diluents was added to every well. The plate was covered and incubated for 1 hour on a shaker. After new washes, diluted Streptavidin-RPE was added to each assay well. Plate was covered and incubated for 30 minutes on a shaker. Finally, after washing, standards were added into wells and plate was covered and incubated the plate for 2 hours at room temperature on a shaker. The plate was read on any immunofluorescence reader.

### 2.4. Statistical Analysis

Statistical analysis was performed by application of Mann-Whitney *U* test using StatView software. The differences were considered significant when *P* was equal or less than 0.05 *P* < 0.05.

## 3. Results

The active study group included 30 children (24 : 6 male : female ratio; 2–15 y.o.: median 7 y.o.) with a clinical diagnosis of persistent CMP allergy expressed as severe recurrent grade II/III anaphylactic episodes [15]. The diagnostic workout showed positive skin prick tests and very high levels of sIgE antibodies to whole CMP (average sIgE to CMP 189 kU/l) and casein protein (178 kU/l) (Table 1). Significant low levels of two macrophage (MIP-1alpha) and monocyte proteins (MCP-1) [15] compared to control group were also observed. MIP levels were 11.4 pg/mL in the active group compared to 26.9 pg/mL in control group (*P* < 0.05); MCP levels was 11.9 pg/mL in the active group compared to 28.7 pg/mL in control group (*P* < 0.05).

All patients successfully completed the clinical follow-up during 12 months with a daily dose of 250 mL of cow's milk. A significant decrease in the degree of the sensitization was observed by either* in vivo* (SPT) or* in vitro* (sIgE) tests or both (Table 1). Most of the CMP adverse reactions were recorded during the first 18 weeks of the protocol. Nonscheduled assistance in the ER was needed in only two patients while the rest of the recorded CMP adverse reactions were properly managed and treated by patients, parents, or guardians at home.

Baseline levels (i.e., prior to CMP desensitization) of PDGF in patients were different compared to the nonallergic children (1170 pg/mL and 501 pg/mL, resp.). The basal levels of VEGF were also significantly different in both groups (253 pg/mL and 108 pg/mL). In both cases, the differences were statistically significant with a *P* < 0.05.

**Figure 1:**
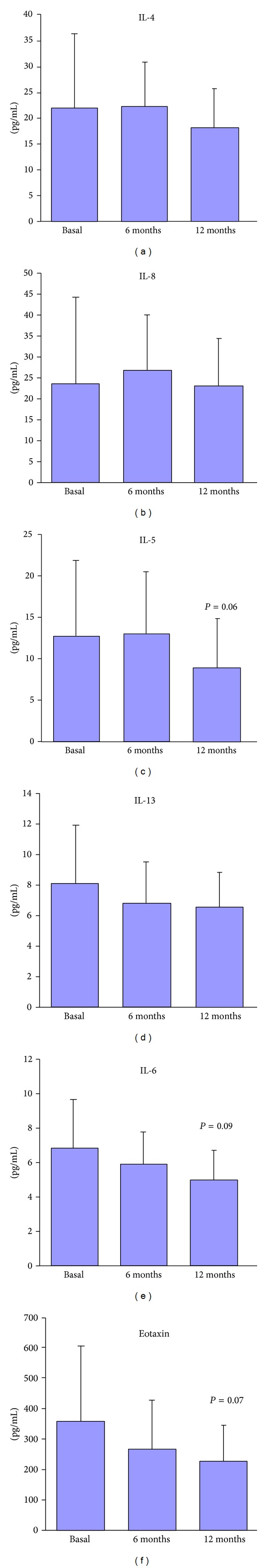
Levels of Th_2_ cytokines in the active group (mean and standard error of patients—*n* = 30—at basal time and 6 and 12 months). In control group there were no differences at any time.

Followup of the levels of both PDGF and VEFG factors were determined and were significantly downregulated (*P* < 0.05) 6 and 12 months after fulfilling the CMP desensitization protocol (Figure 1).

There were not any significant differences in the basal levels of some Th_2_ cytokines between the active and the control group. IL-4 levels were 22.3 pg/mL in active group and 31.5 pg/mL in control group, IL-5 levels were 12.7 pg/mL in active group versus 15 pg/mL in control group, IL-6 levels were 6.9 pg/mL in active group versus 3.0 pg/mL in control group, IL-8 levels were 21.6 pg/mL in active group versus 21.1 pg/mL in control group, and IL-13 levels were 8.2 pg/mL in active group versus 7.0 pg/mL in control group.

No significant differences were either observed in other cytokines tested in both groups (active and control group at baseline time such as IL-2 (20.0 pg/mL in active group versus 17.5 pg/mL in control group), IL-12p40 (381 pg/mL in active group versus 305.5 pg/mL in control group), IL-12p70 (19.2 pg/mL in active group versus 25.0 pg/mL in control group), IFN-*γ* (22.3 pg/mL in active group versus 31.5 pg/mL in control group) and TNF-*α* (17.8 pg/mL in active group versus 14.7 pg/mL in control group), TGF-*β* (3.9 pg/mL in active group versus 3.5 pg/mL in control group), IL-10 (8.7 pg/mL in active group versus 9.0 pg/mL in control group), and IL17 (12.5 pg/mL in active group versus 13.0 pg/mL in control group).

**Figure 2:**
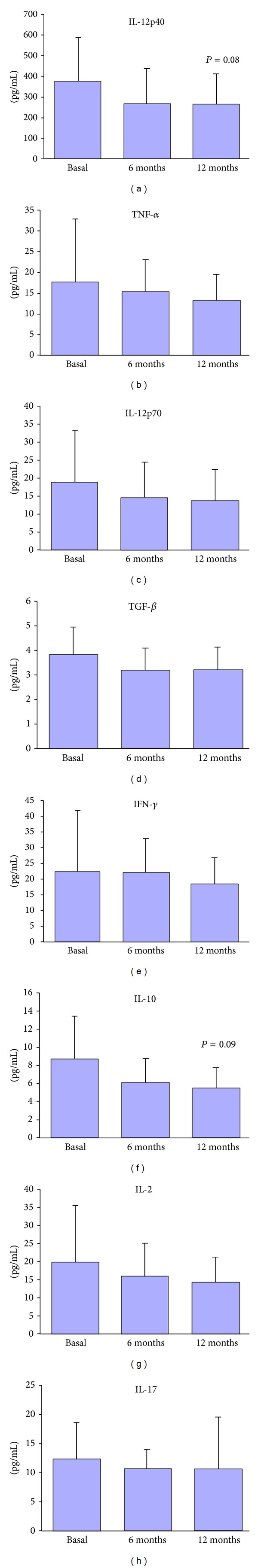
Levels of Th_1_, some T_reg_ and Th_17_ cytokines in the active group (mean and standard error of patients—*n* = 30—at basal time and 6 and 12 months). In control group there were no differences at any time.

As shown in Figure 2, no significant differences were found in IL-4, IL-5, IL-6, IL-8, or IL-13 levels between the basal and 12 months after treatment in the active group. However, differences between basal time and 12 months in active group were near to be significant with *P* < 0.1 with a trend in the case of IL-5 (*P* = 0.06), IL-6 (*P* = 0.09), and eotaxin (*P* = 0.07) (Figure 2).

**Figure 3:**
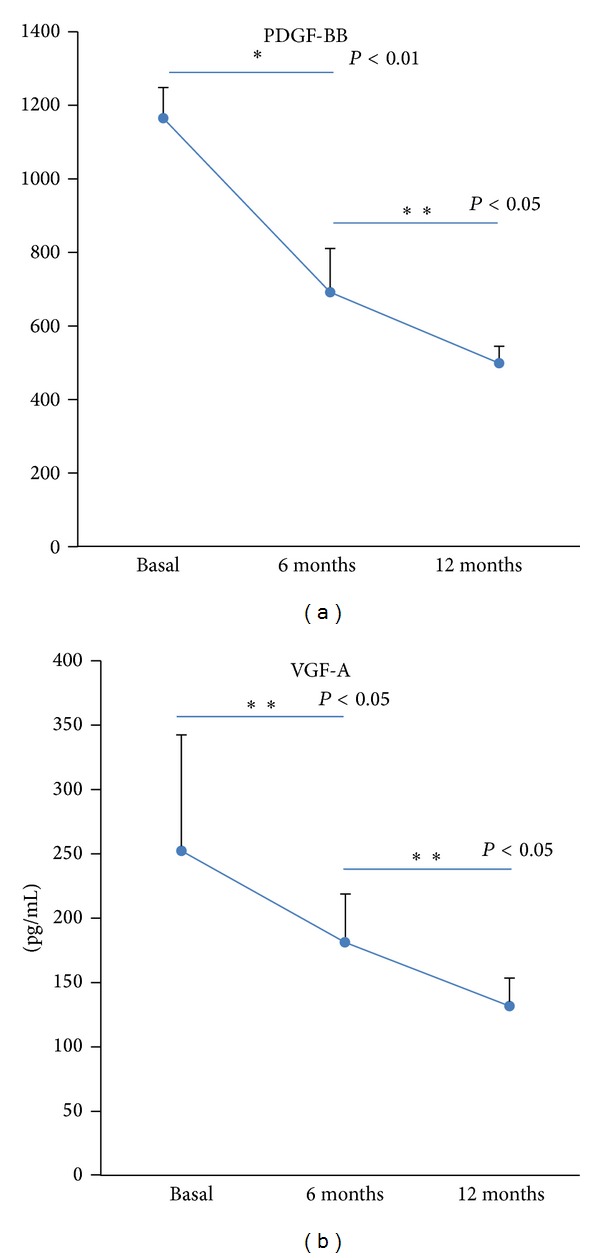
Levels of PDGF-bb and VEGF-A in the active group (mean and standard error of patients—*n* = 30—at basal time and 6 and 12 months). In control group there were no differences at any time.

Similarly, no significant differences were observed in IL-2, IL-12p40, IL-12p70, IFN-*γ* and TNF-*α*, TGF-*β*, IL-10, and IL17 (Figure 3). However, again a tendency to observe a significant difference was observed in IL-12p40 (*P* = 0.08) and IL-10 levels (*P* = 0.09).

Finally, the basal levels of MCP-1 (11.1 pg/ml) and MIP-1*α* (12.1 pg/ml) in children with anaphylaxis were significantly lower than children of the control group (MCP-1 28.4 pg/mL; and MIP-1*α*, 29.8 pg/mL). Levels of these downregulated MCP and MIP-1*α* did not significantly changed one year after oral immunotherapy as previously observed [15].

Remarkably, in all cases, It must be noted that all assays of each of the cytokines were performed under the same conditions and the same plate at the same time, to avoid differences between assays.

## 4. Discussion

Anaphylaxis is known as the most severe allergic reaction [3]. Insect sting, drugs, foods, and even mites are able to trigger human anaphylaxis after activating specific receptors on the mast cell surface. IgE- and non-IgE-dependent mechanisms may cause degranulation of mast cells releasing preformed mediators such as histamine, proteoglycans, or serine proteases and also cytokines and* de novo* lipid mediators.

In early 70s a platelet-activating factor was first linked with allergy and IgE response [18]. This factor, so-called PAF, has been recently defined as an important mediator playing a pivotal role that correlates with the severity of anaphylaxis [19, 20]. In fact, the key treatment of anaphylaxis, epinephrine, has also been associated with human smooth muscle cells after stimulation with PAF [21].

In this study, we were looking for different platelet associated factors, such as PDGF since it can not only be synthesized by activated platelets but also be released by activated macrophages [6]. Interestingly, we have found a significant increase of PDGF levels in the CMP anaphylactic children compared to the CMP nonallergic control subjects. Furthermore, those levels of PDGF were later significantly downregulated after the implementation of a specific clinical intervention as a rapid CMP desensitization protocol, while no changes were found within the control children. This group of CMP anaphylactic patients also presents high levels of VEGF, another potent proangiogenic factor that specifically acts on endothelial cells increasing the vascular permeability. In fact, some early findings provided the evidence that human peripheral blood eosinophils induce angiogenesis [22], the growth of new vessels from preexisting ones, and to some extent VEGF could play an important role in angiogenesis, the subsequent airway remodeling in bronchial asthma [23] and the different phenotypes of asthma [10].

The CMP anaphylactic patients that underwent the two-step CMP rush desensitization had significantly lower levels of PDGF and VEGF-A 6 months after completing the protocol compared to their own baseline determinations. In contrast, the serum levels of the Th_1_, Th_2_, Th_17_, and T_reg_ key cytokines did not significantly change throughout the CMP desensitization. However, there are several subtle changes which, although they are not significantly different, have a clear tendency to be. The nonsignificant changes in IL-5 levels were unexpected, although the *P* value showed a trend (*P* = 0.06). We have highlighted this result as a result that is not statistically significant, but the trend is clear. This result is correlated with the decrease of VEGF and with the decrease of the levels of eotaxin (*P* = 0.06). Similarly, it has been seen with peripheral eosinophils as it has been shown before [24, 25]. However, we did not find any clear tendency in Eosinophilia in our patients. Eosinophilia, which is a marker of allergic processes, especially with respiratory allergy, could increase in cases of occurrence of respiratory sensitization against mites or could decrease in patients with improvement in their atopic dermatitis. All these results would support a cause/effect about the immune-modulatory changes derived from oral immunotherapy. That is why we decided not to include such data in the present paper. All these results would support a cause/effect about the immune-modulatory changes derived from oral immunotherapy.

In case of other important cytokines, we did not see differences. In some cases, such as IFN-*γ*, we firmly think that the assay is not the optimal procedure to detect subtle changes since the detected levels in our samples were extremely low. This should be better informed to avoid spending time and effort in an assay that is better for other molecules.

Downregulation of both VEGF and PDGF [26] after the rapid IgE desensitization, an antigen-specific method that prevents mast cell degranulation and protects patients from food and drug anaphylaxis, [27] has proved as a valuable method to obtain a temporary immunotolerance. This issue along with the additional oral antigen-specific immunotherapy may evidence the a possible mobilization of calcium through into voltage-dependent calcium channels in the desensitization of beta-adrenoceptors in the airway smooth muscle as speculated before [28] and this could be linked to the changes in the levels of PGDF [29]. It could also reflect the result of mast cell degranulation and differential inflammatory cell recruitment, in response to the antigen-specific continuous challenge [27].

Desensitization protocols with drugs have been well described but are considered risky procedures [30] although they may become easier to accomplish when the target cells are found in their refractory period [31] as recently revised [32].

After an accurate diagnosis [33, 34], a rush CMP desensitization protocol is an effective therapeutic intervention for patients with persistent CMP allergy to achieve a clinical tolerance, although immunological tolerance is still unclear [15]. As food allergy probably results from a failure of the correct development of ordinary oral tolerance, the oral route in OIT takes advantage of the cells and immune pathways involved in the induction of oral tolerance. The possible mechanisms of oral food desensitization included increased milk-specific IgG4 with decreased levels of specific IgE followed by decreased activation of mast cells and basophils with their related cytokines. The early downregulation either of IgE or mast cell activating factors could address the success of the oral desensitization therapy, even on those patients with basal high levels of CMP sIgE.

At this time it is unclear if a permanent clinical tolerance could be achieved or it is only transient depending on the maintenance of daily dose [2]. Our data proved that CMP desensitization is a complex process with the potential to induce early immunological changes, not only in the humoral response, that is, sIgE and IGg4, but also in mast cell activity markers, that is, angiogenic factors, that may be of use in the immunological outcome of OIT.

Although this is the first description of the PDGF and VEGF angiogenic factors related to food desensitization in human anaphylaxis, more biological markers are needed to characterize the pathogenesis of this disease.

## Figures and Tables

**Table 1:** Figures of levels of specific IgE against some cow's milk protein fractions in the patient group and control group.

Allergen	Patients group *n* = 30			Control group *n* = 30
	Specific IgE (kU/L)			
	Average	Range	Median	
Whole cow's Milk	189	30–586	146	<0.35
Casein	178	26–544	90	<0.35
*α*-lactalbumin	21	9–34	21	<0.35
*β*-lactoglobulin	16	2–38	7	<0.35
